# Biomarker Profiling with Targeted Metabolomic Analysis of Plasma and Urine Samples in Patients with Type 2 Diabetes Mellitus and Early Diabetic Kidney Disease

**DOI:** 10.3390/jcm13164703

**Published:** 2024-08-10

**Authors:** Maria Mogos, Carmen Socaciu, Andreea Iulia Socaciu, Adrian Vlad, Florica Gadalean, Flaviu Bob, Oana Milas, Octavian Marius Cretu, Anca Suteanu-Simulescu, Mihaela Glavan, Lavinia Balint, Silvia Ienciu, Lavinia Iancu, Dragos Catalin Jianu, Sorin Ursoniu, Ligia Petrica

**Affiliations:** 1 Department of Internal Medicine II, Division of Nephrology, “Victor Babes” University of Medicine and Pharmacy, Eftimie Murgu Sq. No. 2, 300041 Timisoara, Romania; maria.mogos@umft.ro (M.M.); bob.flaviu@umft.ro (F.B.); milas.oana@umft.ro (O.M.); anca.simulescu@umft.ro (A.S.-S.); patruica.mihaela@umft.ro (M.G.); lavinia.balint@umft.ro (L.B.); silvia-ioana.ienciu@umft.ro (S.I.); iuliana.alioani@umft.ro (L.I.); petrica.ligia@umft.ro (L.P.); 2 County Emergency Hospital Timisoara, 300723 Timisoara, Romania; vlad.adrian@umft.ro (A.V.); jianu.dragos@umft.ro (D.C.J.); 3 Centre for Molecular Research in Nephrology and Vascular Disease, Faculty of Medicine, “Victor Babes” University of Medicine and Pharmacy, Eftimie Murgu Sq. No. 2, 300041 Timisoara, Romania; csocaciudac@gmail.com (C.S.); sursoniu@umft.ro (S.U.); 4 Research Center for Applied Biotechnology and Molecular Therapy BIODIATECH, SC Proplanta, Str. Trifoiului 12G, 400478 Cluj-Napoca, Romania; 5 Department of Occupational Health, University of Medicine and Pharmacy “Iuliu Haţieganu”, Str. Victor Babes 8, 400347 Cluj-Napoca, Romania; andreeaiso@gmail.com; 6 Department of Internal Medicine II—Division of Diabetes and Metabolic Diseases, “Victor Babes” University of Medicine and Pharmacy, Eftimie Murgu Sq. No. 2, 300041 Timisoara, Romania; 7 Department of Surgery I—Division of Surgical Semiology I, “Victor Babes” University of Medicine and Pharmacy, Eftimie Murgu Sq. No. 2, 300041 Timisoara, Romania; cretu.marius@umft.ro; 8 Emergency Clinical Municipal Hospital Timisoara, 300079 Timisoara, Romania; 9 Department of Neurosciences—Division of Neurology, “Victor Babes” University of Medicine and Pharmacy, Eftimie Murgu Sq. No. 2, 300041 Timisoara, Romania; 10 Centre for Cognitive Research in Neuropsychiatric Pathology (Neuropsy-Cog), Faculty of Medicine, “Victor Babes” University of Medicine and Pharmacy, Eftimie Murgu Sq. No. 2, 300041 Timisoara, Romania; 11 Center for Translational Research and Systems Medicine, Faculty of Medicine, “Victor Babes” University of Medicine and Pharmacy, Eftimie Murgu Sq. No. 2, 300041 Timisoara, Romania; 12 Department of Functional Sciences III, Division of Public Health and History of Medicine, “Victor Babes” University of Medicine and Pharmacy, Eftimie Murgu Sq. No. 2, 300041 Timisoara, Romania

**Keywords:** diabetic kidney disease, targeted metabolomics, biomarkers, amino acids

## Abstract

**Background**: Over the years, it was noticed that patients with diabetes have reached an alarming number worldwide. Diabetes presents many complications, including diabetic kidney disease (DKD), which can be considered the leading cause of end-stage renal disease. Current biomarkers such as serum creatinine and albuminuria have limitations for early detection of DKD. **Methods**: In our study, we used UHPLC-QTOF-ESI+-MS techniques to quantify previously analyzed metabolites. Based on one-way ANOVA and Fisher’s LSD, untargeted analysis allowed the discrimination of six metabolites between subgroups P1 versus P2 and P3: tryptophan, kynurenic acid, taurine, l-acetylcarnitine, glycine, and tiglylglycine. **Results**: Our results showed several metabolites that exhibited significant differences among the patient groups and can be considered putative biomarkers in early DKD, including glycine and kynurenic acid in serum (*p* < 0.001) and tryptophan and tiglylglycine (*p* < 0.001) in urine. **Conclusions**: Although we identified metabolites as potential biomarkers in the present study, additional studies are needed to validate these results.

## 1. Introduction

Diabetes mellitus (DM) represents a considerable medical challenge due to its high prevalence and associated complications [1]. DKD, a complication of DM, has an immense negative impact on patients’ life expectancy by evolving to end-stage renal disease (ESRD) in almost half of the cases [2]. Although DKD is a frequently encountered condition, the current methods of diagnosis, which include estimated glomerular filtration rate (eGFR) and albuminuria, lack specificity and sensibility for disease onset [3,4].

Albuminuria was the mainstay of DKD identification for a long time. However, recent data indicate that the glomerular tuft is not exclusively affected by long-term hyperglycemia. Emerging evidence points to the proximal tubule as the first affected site in the context of DM. Hence, it is critical to investigate novel biomarkers that may have an effect on these structures [5].

Metabolomics represents a refined and a deeply comprehensive tool that is useful for the investigation of the whole metabolome of a particular person. This technique includes the untargeted and targeted analysis of metabolites encountered in certain biofluids, such as urine and plasma, offering crucial data regarding mechanisms of disease development, drug toxicity, and possibly gene function [6]. This untargeted analysis may be applied to explore the potential metabolic pathways in which the identified metabolites are implicated.

In a step-way fashion, the targeted analysis allows for the quantification of the metabolites previously discovered by untargeted techniques. Metabolites derived from amino acids (AAs), nucleotides, lipids, and sugars are small molecules that can be analyzed by metabolomic techniques [7,8,9].

Amino acids are organic compounds that play a fundamental role in the synthesis of proteins, polypeptides, and other biologically active nitrogenous molecules. The human body is able to synthesize only certain types of amino acids, and the rest of them, such as isoleucine, histidine, lysine, leucine, phenylalanine, tryptophan, methionine, threonine, and valine, are supplied through diet [10]. Diet-derived AAs are metabolized in many tissues, particularly in the liver, intestine, muscle, and kidney. From a renal point of view, AAs are reabsorbed in 99.5% in the proximal tubule, and their dynamics are highly reflective of tubular function [11].

Tryptophan (TRP) is an AA encountered in foods such as milk, beans, fish, and eggs [12]. TRP metabolism follows three major metabolic pathways that are under the control of gut microbiota: (1) the serotonin pathway, which results in the formation of serotonin derivatives via tryptophan hydroxylase; (2) the indole pathway, in which TRP is broken down to form indole derivatives under the action of the intestinal microbial community; and (3) the kynurenine (KYN) pathway, which utilizes indoleamine 2,3-dioxygenase (IDO) to produce kynurenine derivates [13,14,15]. Studies conducted by Chou et al. and Wu et al. revealed that low serum TRP concentrations are associated with a rapid decline in eGFR and that a higher KYN/TRP ratio is linked to macroalbuminuria [2,16].

Taurine is a sulfonic amino acid located in the cerebral, muscular, cardiovascular, and gastrointestinal systems and is endogenously synthesized from cysteine. In the kidney, taurine is involved in multiple cellular functions, such as ion absorption (efflux from renal cells requires the presence of Na^+^ and Cl^−^) and renal vascular endothelial function (high levels of taurine lead to increased serum levels of nitric oxide and NO synthase activity); the renin-angiotensin-aldosterone system is also affected by taurine, which yields high endothelin levels [17]. Bergstrom et al. revealed reduced taurine concentrations in the plasma pf patients with chronic kidney disease [18]. Moreover, other studies pointed out significantly decreased serum taurine levels in patients with DKD [19,20,21,22].

Carnitines are derived from amino acids and include L-carnitine and its acylated forms, such as acetyl-L-carnitine and propionyl-L-carnitine. Carnitines can be found in foods and also may be secreted endogenously in humans. Belay B et al. stated that carnitines play a critical role in energy production by transporting long-chain fatty acids into mitochondria so that they can be oxidized to produce energy [23]. In the kidney, Van der Kloet et al. observed that under certain conditions, such as a diabetic state, reabsorption of acylcarnitine is poor, thus they are eliminated through urine [24,25].

Glycine is an amino acid that participates in multiple metabolic pathways. It is synthesized in the renal and hepatic systems from threonine, choline, and serine via the threonine dehydrogenase pathway, the generation of sarcosine, and the serine hydroxymethyltransferase pathway, respectively [26]. Although the mechanism by which glycine acts in patients with T2DM is not very clear, the authors consider that this is related to low levels of serine. Also, the excessive metabolism of free fatty acids (FFAs) and BCAAs (branched-chain amino acids) leads to low levels of glycine [27]. Tiglylglycine is an intermediate product of the catabolism of isoleucine, and it is less studied in the literature. Two recent reports describe a high secretory clearance of this metabolite among patients with DKD [28,29].

Renal disease in T2DM is associated with increased morbidity and mortality. Although large studies offer insights on the evolution of DKD, early biomarkers are needed to elaborate algorithms and improve the stratification of individuals with renal disorders [30]. The current study represents an extension of a previous study where we used metabolomic analyses of serum and urine samples from T2DM patients [31]. The aim of the study is to use targeted analyses to identify novel biomarkers that can predict early renal impairment in DKD.

## 2. Materials and Methods

### 2.1. Patients and Compliance with Ethical Standards

In this cross-sectional pilot study, we enrolled 140 T2DM patients between June 2021 and April 2022 from the Department of Nephrology and Department of Diabetes and Metabolic Diseases “Pius Brinzeu” County Emergency Hospital Timisoara. As an inclusion criterion, we selected patients with long-standing T2DM, with a duration of more than five years. Patients with T2DM with poor control of diabetes (HbA1c > 10%), active infections, neoplasia, glomerular disease, end-stage renal disease, and T1DM were excluded from the study. At the time of screening, all patients received treatment with angiotensin converting enzyme inhibitors or angiotensin receptor blockers, oral antidiabetic agents, insulin, and/or statins. Our research was based on a pilot study that involved the collection of serum and urine samples from 110 eligible subjects, including 90 T2DM patients (group P) and 20 healthy control subjects. Based on UACR, we divided the 90 T2DM patients in 3 subgroups (30 normoalbuminuria < 30 mg/g subgroup P1, 30 microalbuminuria 30–300 mg/g subgroup P2, and 30 macroalbuminuria > 300 mg/g subgroup P3) and 20 healthy controls (group C) [31].

Considering that the group of investigated patients is the same as the group described in the previous article, the representative demographic and clinical parameters can be found in Table 1 of our first study [31].

The protocol of the study was approved by the Ethics Committee for Scientific Research of “Victor Babes” University of Medicine and Pharmacy Timisoara (no. 28/02.09.2020) and the Ethics Committee for Scientific Research of “Pius Brinzeu” County Emergency Hospital Timisoara (no. 296/06.04.2022). We obtained written consent from all patients enrolled in this study.

### 2.2. Chemicals and Reagents

For the targeted analysis of this study, we used the following chemicals and regents: HPLC-grade formic acid and HPLC/MS-grade formic acid and acetonitrile. The standard biomarkers used include the following: L-tryptophan, O-acetyl-L-carnitine hydrochloride, kynurenic acid, taurine, tiglylglycine, glycine from amino acid standard H, and creatinine > 98%. Doxorubicin hydrochloride was used as an internal standard. Other reagents include LC-MS-grade MeOH, MeCN, and formic acid, which were purchased from Fisher Scientific. Ultra-pure water was purified using a Milli-Q water system. Instruments used in this study included a vortex mixer, Minicentrifuge Eppendorf (Thermo Fisher Scientific, Waltham, MA, USA), and UPLC-Q-TOF/MS.

### 2.3. Sample Preparation

For the samples collected from fasting patients, confidential numerical codes were used to preserve their identity. Venipuncture was the method used to collect blood to obtain serum. The blood samples were put in sterile vacutainers without anticoagulant, and urine samples were placed in sterile vials. The samples were held at −80 °C until analysis. A volume of 0.8 mL of a mix of pure HPLC-grade methanol and acetonitrile was added to each 0.2 mL of serum and 0.2 mL urine, separately. In each case, the mixture was vortexed to precipitate proteins, ultrasonicated, and stored at −20 °C for 24 h to increase the amount of protein precipitated. The supernatant was collected after centrifugation and filtered through nylon filters. Finally, it was placed in glass microvials and introduced into the autosampler of the UHPLC before injection. The supernatant was transferred to an autosampler vial for HPLC-MS analysis. Quality control (QC) samples were also obtained and used as representative generic samples that were injected at the beginning and end of the experiment and at every 10th injection while analyzing the study samples.

### 2.4. UHPLC-QTOF-ESI^+^-MS Analysis

The metabolomic profiling was performed with UHPLC-QTOF-ESI+-MS using a ThermoFisher Scientific UHPLC Ultimate (Bruker GmbH, Berlin, Germany) 3000 instrument equipped with a quaternary pump, Dionex delivery system, and MS detection equipment with MaXis Impact (Bruker Daltonics, Billerica, MA, USA). Details about this method are described in our previous study [31].

### 2.5. Data Processing and Statistical Analysis

In our first study, we performed untargeted analysis for the same groups (P and C) [31].

Initially, multivariate analysis was performed to indicate the differences between the 2 groups (C group versus P group) using fold change, PCA, and PLSDA score plots, including VIP values. Volcano plots were generated with the log2 fold change values and Bonferroni-adjusted *p* values. Here, *p* < 0.05 was defined as statistically significant.

In order to evaluate the particularities of each metabolite, we performed receiver operating characteristic (ROC) analysis. Metabolites with AUC values higher than 0.8 met the criteria to be considered potential biomarkers in future studies because they have a high predictive effect on disease.

One-way ANOVA univariate analysis aimed to discriminate between the controls and the subgroups of patients (P1, P2, P3). PCA and PLSDA score plots including VIP values, cross validation parameters, and the mean decrease accuracy scores obtained using random forest analysis were generated. For the statistical analysis of targeted metabolites, matrices were selected that included the five metabolites mentioned above (*m*/*z* values versus MS peak intensity obtained as .csv files) [31].

From untargeted metabolomics analysis, we selected seven specific molecules. To compare the various subgroups of patients we used mass spectrometry (MS) peak intensities. Specific biomarkers selected by the untargeted metabolomics were calculated using mean values of peak intensity (PI) and their standard deviations (SD). For quantitative analysis, pure standards were determined using calibration curves.

### 2.6. Metabolite Identification

Untargeted metabolomic analysis identified five significant metabolites that can be considered as putative biomarkers for differentiation between subgroups. For multivariate and univariate analysis, we utilized the Metaboanalyst 5.0 platform in order to perform the statistical analysis. Moreover, the platform mentioned above was used to identify and describe the major metabolic pathways and the way these metabolites were affected during disease onset.

Platforms such as the Human Metabolome Database, PubChem, and Lipidmaps helped us with chemical information about metabolites taking into account a deviation of the *m*/*z* value of 0.05. By associating the exact number (*m*/*z*) with the ionization method and differentiating the primary and secondary mass spectra information, metabolites with the theoretical fragments of the HMDB search results were used to validate the identification of metabolites.

### 2.7. Quantitative Evaluation

The following stock solutions were used for calibration and quality control of the five potential biomarkers: 1 µM L-tryptophan, 1 µM L-acetylcarnitine, 1 µM kynurenic acid, 1 µM taurine, 2 µM glycine, and 2 µM creatinine. The solutions were diluted in methanol: acetonitrile in order for use in external calibration. In parallel, standard solutions were added to QC deproteinated samples.

The UHPLC-QTOF-ESI^+^-MS method evaluates the limit of detection (LOD) and limit of quantification (LOQ) and was validated according to the “Guidance for Industry-Bioanalytical Method Validation”. Two calibration curves were generated: (1) an external standard calibration curve and (2) an internal standard curve. The mean peak area of three replicate measurements at each concentration was calculated.

The LOD was the lowest concentration of analyte in the test sample that can be discriminated from zero with a signal/noise ratio ≥ 10, and the LOQ was the lowest concentration of analyte that can be evaluated with an acceptable repeatability and trueness (signal/noise ratio ≥ 10 and SD values ≤ 40%).

## 3. Results

### 3.1. Untargeted Multivariate and Univariate Analyses

The untargeted multivariate and univariate UHPLC-QTOF-ESI^+^-MS analyses performed in our previous study [31] provided seven metabolites that could be considered potentially significant biomarkers in early DKD.

Using data from UHPLC-QTOF-ESI^+^-MS analysis, we determined the matrices representing retention times (RT), the mass-to-charge ratio (*m*/*z*), and the area under the curve (AUC) for the seven targeted metabolites in order to perform the statistical analysis shown in Table 1.

The metabolites with AUC values above 0.600 included kynurenic acid, glycine, and L-tryptophan in serum and glycine, creatinine, taurine, tiglylglycine, kynurenic acid, L-acetylcarnitine, and L-tryptophan in urine.

To evaluate the effect of albuminuria (ranging from normal to macroalbuminuria) on the differentiation of the biomarkers, the one-way ANOVA algorithm was applied to compare the group C versus subgroups P1, P2, and P3.

The untargeted analysis allowed the discrimination of six metabolites between subgroups P1 versus P2 and P3, based on one-way ANOVA and Fisher’s LSD as displayed in Table 2A,B.

Statistical data obtained by the methods mentioned above point out metabolites considered for the targeted analysis: glycine, creatinine, taurine, kynurenic acid, L-acetylcarnitine, and L-tryptophan in serum and glycine, creatinine, taurine, tiglylglycine, kynurenic acid, L-acetylcarnitine, and L-tryptophan in urine. Their discrimination between subgroups and their differences expressed in MS peak intensities are represented in Table 2A,B.

According to the data presented above, serum kynurenic acid, L-acetylcarnitine, and creatinine showed higher *f* values (above 1.3). FDR values ranged from 0.035 to 0.645, and the *p* values were not significant (*p* > 0.1) except those for kynurenic acid and creatinine. The significance of the discrimination between groups and subgroups is revealed by Tukey’s HSD post hoc test, which revealed the highest differences between C-P1, C-P2, and C-P3 as well as between P1-P3 and P2-P3 for these metabolites.

Urine glycine showed highest *f* values (above 100). For the other metabolites, the *f* values ranged from 5.988 to 0.391. The *p* values were not significant (*p* > 0.1), except those for glycine, taurine, and tiglylglycine. The FDR values ranged from 0.0003 to 0.445. The significance of the discrimination between groups and subgroups is also revealed here with the Tukey’s HSD post hoc test results, showing the highest differences between C-P1, C-P2, and C-P3 for these metabolites as well as between P1-P2 and P1-P3.

### 3.2. Multivariate Analysis for Targeted Metabolites: Fold Change, p Values, and AUC

#### 3.2.1. Calibrations and Validation Parameters

Determination of linear ranges (calibration curves and equations including R^2^ values), LODs, and LOQs of each standard are given in Table 3. The correlation coefficients (R^2^) were higher than 0.898 for all standards in their linear range, showing good linear relationships within linear ranges. All the LOD values were in the range of 0.2–4 μM, and LOQ values were in the range of 0.8–5.5 μM.

The validation of the LC-MS method for the quantitative evaluation of metabolites was done using controlled additions of the internal standard (DOXO) and each of the five pure standards to QC extracts.

Briefly, 0.2 mL of each of the eight standard solutions (50 mM creatinine and glycine, 25 mM taurine and L-tryptophan, 5 mM kynurenic acid, L-Acetylcarnitine) and 3.4 mM of internal standard DOXO were added to the same volume of QC extracts (0.3 mL). Table 4 shows the initial concentrations of metabolites after mixing with the QC extract and the measured concentrations after LC-MS analysis. The recovery percentage was calculated as a measure of the method’s reproducibility.

#### 3.2.2. Quantitative Evaluation

The quantitative evaluation based on the curve equations for each biomarker in serum and urine is presented in Table 5 and Table 6. The mean values and standard deviations (*±SD*) of serum concentrations (µM) of the potential biomarkers targeted in this study are presented for groups C and subgroups P1, P2, and P3.

The graphically differences (expressed in MS peak intensities) among groups C, P1, P2, and P3 for each potential biomarker targeted in serum and urine, considering their original concentrations and the normalized concentrations based on median values, are presented in the Supplementary File (Figures S1 and S2).

For a better understanding of the discriminations and to find the best prediction among the proposed biomarkers, the VIP scores derived from PLSDA analysis and the mean decrease accuracy (MDA) scores derived from random forest analysis are also described in the Supplementary File (Figures S3 and S4).

Calibration curves of the selected putative biomarkers creatinine (from blood serum and urine), acetylcarnitine, kynurenic acid, glycine, taurine, and tryptophan can be found in the Supplementary File (Figure S5).

Subsequently, we correlated the data with references from the HMDB (Table S1 presented in the Supplementary File).

## 4. Discussion

In this study, we performed a comparative targeted metabolomic analysis of blood and urine samples from T2DM patients and healthy subjects, with a special focus on the normoalbuminuric subgroup. The goal of this research was to establish that the potential biomarkers that were initially selected using untargeted analysis in our previous study [31] can be confirmed by multivariate and univariate analyses (ANOVA) as well as quantitative evaluation.

In the targeted analysis, calibration and validation curves were employed in order to evaluate the concentration and the way in which each targeted biomarker undergoes changes during the evolution of a disease. From the previous study, we selected the following biomarkers with potential roles in diagnosis of incipient DKD: tryptophan, kynurenic acid, taurine, L-acetylcarnitine, glycine, and tiglylglycine. We found several metabolites that can be considered putative biomarkers in early DKD, including glycine and kynurenic acid in serum and tryptophan and tiglylglycine in urine.

### 4.1. TRP Metabolic Pathway and Its Role in DKD

In our study and according to our statical analysis (Table S2 in the Supplementary File), we detected that TRP had a tendency to decrease progressively in accordance with the evolution of albuminuria. By contrast, in urinary samples, we found that normoalbuminuric patients had low TRP concentrations in group P1 versus group C as well as in subgroups P2 and P3; thus, TRP can be considered an important early DKD biomarker. These findings are in agreement with prior studies where Chou et al., Faheem et al., and Wu et al. reveal low serum concentrations of TRP associated with a reduction in eGFR as well as macroalbuminuria [2,16,32]. When compared to the HMDB, the concentrations of TRP vary between 40 and 90 µM in blood and between 3 and 6 µM/mM creatinine in urine samples. In addition, kynurenic acid concentrations vary between 0.03 and 0.007 < 5 µM in serum and between 1 and 1.6 µM/mM creatinine in urine samples. In addition, Chou et al. reported TRP concentrations that were < 44.2 µmol/L, and Wu et al. reported that TRP < 46.75 µmol/L in macroalbuminuric patients.

TRP is an amino acid that contributes to generating proteins. TRP has complex biological effects due to numerous metabolites implicated in many metabolic pathways. Tryptophan metabolism follows three major pathways: the serotonin pathway in which TRP is catalyzed with TRP hydroxylase1 (TPH1) in the enterochromaffin cells to produce peripheral 5-HT that participated in the regulation of gastrointestinal functions; the indole pathway, where TRP is directly metabolized to indole and indole derivates via intestinal flora; and the kynurenine pathway in which TRP is metabolized by 3 enzymes, including IDO1, IDO2, and TRP 2,3-dyoxygenase (TDO), to produce formyl kynurenine (FKYN) that catalyzes formilase to generate kynurenine and other bioactive metabolites, such as kynurenic acid, picolinic acid, nicotinamide adenine dinucleotide, and xanthurenic acid [12]. According to data presented by Mohib et al. and Stone TW et al., there is an excessive accumulation of the IDO1 enzyme in the kidney tissues, which can lead to the assumption that the changes in the activity of the enzymes in the kynurenine pathway could contribute to the pathogenesis of DKD [33,34].

As a metabolite of TRP, serum kynurenic acid displayed similar functions as TRP with a decreased trend in serum from the C group to P subgroups, but significance was not achieved in the urine samples.

### 4.2. Assessment of Taurine as a Potential Biomarker in DKD

In this article, we reported a slight decrease in taurine concentrations in blood samples from the control group to normoalbuminuric group and a slight elevation in subgroups P2 and P3, separately. In contrast, in urine samples, we found out that concentrations of this metabolite do not vary between group C and subgroups P1, P2, and P3, separately. Some of this information can be related with data from the literature where Bergstrom et al. describe reduced taurine concentrations in the plasma of patients with CKD [18]. Regarding patients with DKD, the authors of other studies highlight the same decline in serum levels of taurine [19,20,21,22]. Also, in a recent review, a substantial decrease in serum taurine was noted in diabetic patients when compared to the control group [35]. As a less studied metabolite, we could not find data on urine concentrations of taurine. As stated by the HMDB, the normal range is 40–80 µM in serum and 25–90 µM/mM creatinine in urine samples.

Taurine is a sulfonic amino acid found in great quantity in almost all tissues and cells of animals and humans [36], and it is produced in the liver from methionine or cysteine. This metabolite is involved in many physiological processes. For example, taurine has implication in glucose homeostasis by reducing oxidative stress, inflammation, and increasing insulin sensitivity and secretion [37].

The diffusion of taurine takes place via a sodium-dependent transporter [38]. In addition to sodium, renal epithelia require chloride or bromide to assimilate taurine [39]. The mechanism of sodium and chloride explains that Na^+^ moves into cells using a gradient and subsequently is removed from the cell by the Na^+^K^+^-dependent ATPase. Taurine transport is inhibited by β amino acids and GABA (gamma-aminobutyric acid) and is membrane surface specific. In renal tubules, taurine behaves differently depending on the location: in the proximal tubule the maximal uptake is on the apical surfaces, while the uptake of this metabolite is at the level of basolateral surface in distal tubule [40].

The mechanism described above shows that taurine is not involved in an adaptive renal response but is dependent on specific ions to participate in passive diffusion. Also, taurine interacts with glucose. In the renal system, taurine appears to reduce glucose using an Na^+^-dependent mechanism that can potentially determine glucosuria. It was presumed that the presence of taurine in the renal tubules compete for sodium, which leads to a reduction in glucose. As a consequence, taurine may have impact on the intracellular or transcellular movement of glucose [17].

Moreover, taurine represents a substrate for mitochondrial tRNA. A study by Fakruddin et al. demonstrated an activation of cellular oxidative stress by mitochondrial dysfunction due to a deficit in taurine [41]. Also, it can suppress oxidative stress by diminishing excess levels of calcium, increasing antioxidant glutathione, and inhibiting ROS (reactive oxygen species) [21,42,43].

### 4.3. Association between Acetylcarnitine and the Presence of DKD

Our paper describes high serum concentrations of L-acetylcarnitine in subgroup P1 versus group C and subgroups P2 and P3, separately; however, in urine samples, this metabolite did not achieve significance between subgroups. According to the HMDB, the normal range is 5–7 µM in serum and 1–3 µM/mM creatinine in urine samples. L-Acetylcarnitine, the acetylated form of the amino acid derivate l-carnitine, is a small molecule that fulfills essential roles in intermediate metabolism. It is implicated in cellular energy, which, through β-oxidation, transfers fatty acids from the cytosol into mitochondria. Also, carnitines control the activity of some mitochondrial enzymes involved in the tricarboxylic cycle (TCA), urea cycle, and gluconeogenesis [44]. In several studies, it was documented that the level of serum acetylcarnitines increases with the depletion of renal function in patients with CKD [45,46]. Regarding the significance of this metabolite in patients with T1DM and T2DM, the studies conducted by Van der Kloet et al. and Gunther et al. show the associations between serum and urinary acetylcarnitines and the risk of diabetes development [24,47].

### 4.4. Glycine and Its Metabolite Tiglylglycine—Involvement in DKD

Regarding the concentration of glycine determined in this research, we observed a downward trend in glycine levels in blood and urine samples, and these results are consistent with data in the literature where Barrios et al. and Yatzidis et al. found strong associations between low levels of glycine and a decrease in eGFR [48,49]. Moreover, Floegel et al. and Sekher et al. compared patients without diabetes and those with diabetes and discovered reduced concentrations of glycine [50,51]. Glycine is a metabolite synthesized from serine. Although the mechanism by which glycine acts in patients with T2DM is not very clear, the authors consider that this is related to low levels of serine. Also, increased metabolism of free fatty acids (FFAs) and BCAAs can induce low levels of glycine [27].

In agreement with the HMDB, the normal range for glycine is 120–450 µM in serum and 106–135 µM/mM creatinine in urine samples, whereas the normal range for tiglylglycine is 0.1–7 µM/mM creatinine in urine samples. Of interest, this metabolite was not detected in blood samples.

Tiglylglycine, a metabolite of glycine, has the same characteristics as glycine. We could not identify this metabolite in plasma, but we detected progressively decreasing levels from group C to subgroup P3Statistical analysis revealed low concentrations in urine in the P3 subgroup as compared to our previous study, where we revealed increased values of this metabolite in the P3 subgroup. Tiglylglycine is less studied in the literature, but a few recent reports describe differences in this metabolite between DKD and non-diabetic CKD patients [29,46,52].

### 4.5. From Metabolic Studies to Clinical Practice

The biomarkers mentioned above may have implication in clinical practice. Studies performed on glycine administration indicate improvements in eGFR and DM [49,51,53]. There are contradictions regarding supplementation with taurine. For example, Hansen SH et al. and Harada H et al. demonstrated that the administration of taurine improves high levels of glycemia and reduced sensitivity to insulin in diabetic rats, while Chauncey et al. reported that supplementation of taurine does not have effect on glucose in patients with T2DM [54,55,56]. Furthermore, regarding tryptophan, Inubushi et al. stated that L-tryptophan has an effect on serum glucose and insulin levels after oral glucose administration and restricted glucose assimilation from the intestine in diabetic rats [57].

### 4.6. Other Potential Metabolites in Patients with DM

Alternative biomarkers, such as cystatin C (CysC), neutrophil gelatinase-associated lipocalin (NGAL), kidney injury molecule-1 (KIM-1), N-acetyl-β-(D)-glucosaminidase (NAG), ceramides, apoB, and biomarkers of oxidative stress and inflammation, have been evaluated for the diagnosis of DKD, and many studies have shown encouraging results [58].

## 5. Conclusions

In the present study, we incorporated information about serum and urine metabolites using LC-UHPLC techniques in order to reveal the differences between controls and subgroups of patients with DKD, with particular interest on the normoalbuminuric subgroup. Our results showed several metabolites that can be considered putative biomarkers in early DKD, such as glycine and kynurenic acid in serum and tryptophan and tiglylglycine in urine.

This research has its limitations. First, this is a cross-sectional study, which does not allow the establishment of causality between our findings and clinical parameters. Second, variability in glycemic control could have introduced a bias in the interpretation of data. Third, lipid metabolism variables were not utilized in correlations.

However, our study has its strength, which resides in the documentation of a particular metabolomics profile in serum and urine in patients with T2DM that is highly indicative of early renal involvement within the confines of DKD.

The candidate metabolites from this study need to be validated in larger studies, and their implications in different mechanisms of DKD requires further research.

## Figures and Tables

**Table 1:** Retention times and AUC values for the metabolites found in serum and urine used to identify the differences between the healthy group (controls) and pathological DKD group (P).

*m*/*z*	Identification	RT (min)	Plasma AUC	Urine AUC
76.0815	Glycine	0.9	0.868	0.926
114.0983	Creatinine	0.7	0.512	0.637
125.1042	Taurine	1.6	0.602	0.525
190.0625	Kynurenic acid	11.4	0.787	0.591
204.1369	L-Acetylcarnitine	1.	0.556	0.550
205.1068	L-Tryptophan	2.8	0.618	0.788
158.106	Tiglylglycine	10.7	-	0.684

**Table 2:** (**A**) Statistical data obtained with one-way ANOVA including the Tukey’s HSD post hoc test for the metabolites targeted in serum of controls versus P1–P3 subgroups: *f* value, *p* value, FDR, and significance of differences according to post hoc Tukey’s HSD. (**B**) Statistical data obtained with one-way ANOVA including the Tukey’s HSD post hoc test for the metabolites targeted in urine of controls versus P1-P3 subgroups: *f* value, *p* value, FDR, and significance of differences according to post hoc Fisher’s LSD.

A					
*m*/*z*	Identification	*f* Value	*p* Value	FDR	Tukey’s HSD
76.0815	Glycine	0.624	0.601	0.645	P1-C; P2-C; P3-C; P2-P1; P3-P1; P3-P2
114.0983	Creatinine	2.188	0.094	0.281	P1-C; P2-C; P3-C; P2-P1; P3-P1; P3-P2
125.1042	Taurine	0.984	0.403	0.605	P1-C; P2-C; P3-C; P2-P1; P3-P1; P3-P2
190.0625	Kynurenic acid	4.395	0.006	0.035	P1-C; P2-C; P3-C; P2-P1; P3-P1; P3-P2
204.1369	L-Acetylcarnitine	1.303	0.277	0.555	P1-C; P2-C; P3-C; P2-P1; P3-P1; P3-P2
205.1068	L-Tryptophan	0.556	0.645	0.645	P1-C; P2-C; P3-C; P2-P1; P3-P1; P3-P2
**B**					
***m*/*z***	**Identification**	***f* Value**	***p* Value**	**FDR**	**Tukey’s HSD**
76.0815	Glycine	104.260	0.0005	0.0003	C-P1; C-P2; C-P3; P2-P1; P1-P3; P2-P3
114.0983	Creatinine	0.391	0.759	0.759	C-P1; P2-C; P3-C; P2-P1; P3-P1; P2-P3
125.1042	Taurine	2.316	0.080	0.140	C-P1; C-P2; C-P3; P1-P2; P3-P1; P3-P2
158.1060	Tiglylglycine	2.910	0.038	0.089	C-P1; C-P2; C-P3; P2-P1; P3-P1; P3-P2
190.0625	Kynurenic acid	1.916	0.131	0.184	C-P1; C-P2; P3-C; P1-P2; P3-P1; P3-P2
204.1369	L-Acetylcarnitine	1.032	0.382	0.445	C-P1; C-P2; C-P3; P2-P1; P1-P3; P2-P3
205.1068	L-Tryptophan	5.988	0.001	0.003	P1-C; P2-C; P3-C; P1-P2; P3-P1; P3-P2

**Table 3:** Validation parameters (linear range, curve equation, R^2^, LOD, and LOQ) for each of the seven molecules selected as potential biomarkers.

Name	Linear Range μM	Curve Equation	R^2^	LOD (μM)	LOQ (μM)
Glycine	10–200	y = 596.72x − 963.05	0.993	0.2	0.8
Creatinine—serum	5–25	y = 2693.3x − 254.5	0.994	0.5	1.0
Creatinine—urine	200–2000	y = 2765.8x − 73120	0.993	0.5	1.0
Taurine	5–25	y = 1479.6x + 543.2	0.996	0.5	1.0
Kynurenic acid	0.1–2	y = 24309x + 355.37	0.999	0.08	0.1
L-Acetylcarnitine	1–5	y = 36813x − 3879.2	0.994	0.2	0.8
L-Tryptophan	3–50	y = 820.31x − 981.3	0.998	0.8	1.00

**Table 4:** The recovery percentage (%) calculated from the measured concentrations of internal standard (IS) and each metabolite (pure standard) compared to their initial concentrations after addition to the QC extract.

Metabolite	Initial Concentration (µM)	Measured Concentration (µM)	Recovery (%)
Glycine	20	17.5	87.5
Creatinine	20	18.2	91.0
Taurine	10	8.55	85.5
Kynurenic acid	2	1.65	82.5
L-Acetylcarnitine	2	1.85	92.5
L-Tryptophan	10	9.15	91.5
IS (DOXO)	1.4	1.25	89.3

**Table 5:** The mean values and standard deviations (*±SDs*) of serum concentrations (µM) of the potential biomarkers targeted in this study for groups C and subgroups P1, P2, and P3.

Molecule	Control (n = 20)		P1 (n = 30)		P2 (n = 30)		P3 (n = 30)	
	Mean	*±SD*	Mean	*±SD*	Mean	*±SD*	Mean	*±SD*
Glycine	221.32	*39.84*	196.24	*39.25*	188.56	*43.37*	214.62	*47.22*
L-tryptophan	56.59	*10.19*	51.38	*10.28*	49.53	*11.39*	54.51	*11.99*
Kynurenic acid	5.81	*1.05*	4.80	*0.96*	4.50	*1.04*	4.32	*0.95*
Taurine	86.04	*15.49*	85.98	*17.20*	75.99	*17.48*	80.34	*17.67*
L-Acetylcarnitine	5.52	*0.99*	5.73	*1.15*	5.41	*1.24*	5.58	*1.23*
Creatinine (S)	75.26	*13.55*	74.20	*14.84*	65.67	*15.10*	105.06	*23.11*

**Table 6:** The mean values and standard deviations (*±SDs*) of urine concentrations (µM) of the potential biomarkers targeted in this study for groups C and subgroups P1, P2, and P3.

Molecule	Control (n = 20)		P1 (n = 30)		P2 (n = 30)		P3 (n = 30)	
	Mean	*±SD*	Mean	*±SD*	Mean	*±SD*	Mean	*±SD*
Glycine	114.89	*20.68*	87.36	*17.47*	87.43	*20.11*	111.39	*24.51*
Tiglylglycine (μM glycine units)	22.08	*3.97*	17.45	*3.49*	16.69	*3.84*	29.77	*6.55*
L-tryptophan	7.63	*1.37*	12.14	*2.43*	9.03	*2.08*	16.64	*3.66*
Kynurenic acid	0.34	*0.06*	0.42	*0.08*	0.35	*0.08*	0.59	*0.13*
Taurine	7.6	*1.37*	7.58	*1.52*	6.65	*1.53*	10.72	*2.36*
L-Acetylcarnitine	0.78	*0.14*	0.65	*0.13*	0.63	*0.14*	0.56	*0.12*

## Data Availability

The data that support the findings of this study are available from the corresponding author upon reasonable request.

## Supplementary Materials

The following supporting information can be downloaded at: https://www.mdpi.com/article/10.3390/jcm13164703/s1, Figure S1: Graphic representation of the differences (expressed in MS peak intensities) between the groups C, P1, P2 and P3, for each of the serum potential biomarkers. The original and normalized (sample normalization by median values) are presented. Figure S2: Graphic representation of the differences (expressed in MS peak intensities) between the groups C, P1, P2 and P3, for each of the urine potential biomarkers. The original and normalized (sample normalization by median values) are presented. Figure S3: (A) VIP Scores plot for the serum samples from groups C *vs* subgroups P1, P2, P3 of DKD patients, (B) Random Forest analysis: Mean decrease accuracy (MDA) scores showing the ranking of best predicted biomarkers in serum samples. Figure S4: (A) VIP Scores plot for the urine samples from groups C *vs* subgroups P1, P2, P3 of DKD patients, (B) Random Forest analysis: Mean decrease accuracy (MDA) scores showing the ranking of best predicted biomarkers in urine samples. Figure S5: Calibration curves for the selected putative biomarkers: creatinine (from blood serum and urine), Acetylcarnitine, Kinurenic acid, Glycine, Taurine, Tryptophan. Table S1: Metabolites range. Table S2: Statiscal analysis.
