# Supplementary material for: Biomarker Profiling with Targeted Metabolomic Analysis of Plasma and Urine Samples in Patients with Type 2 Diabetes Mellitus and Early Diabetic Kidney Disease
Source: J Clin Med. 2024 Aug 10;13(16):4703. doi: 10.3390/jcm13164703 (PMC11355042; doi:10.3390/jcm13164703)
Supplement: Supplementary file 1 [file jcm-13-04703-s001.zip › jcm-3095334-supplementary.pdf]

Fig. S1 shows graphically the differences (expressed in MS peak intensities) between the groups C, P1, P2 and P3, for each potential serum biomarker targeted in blood serum, considering their original concentrations and the normalized ones by median values.

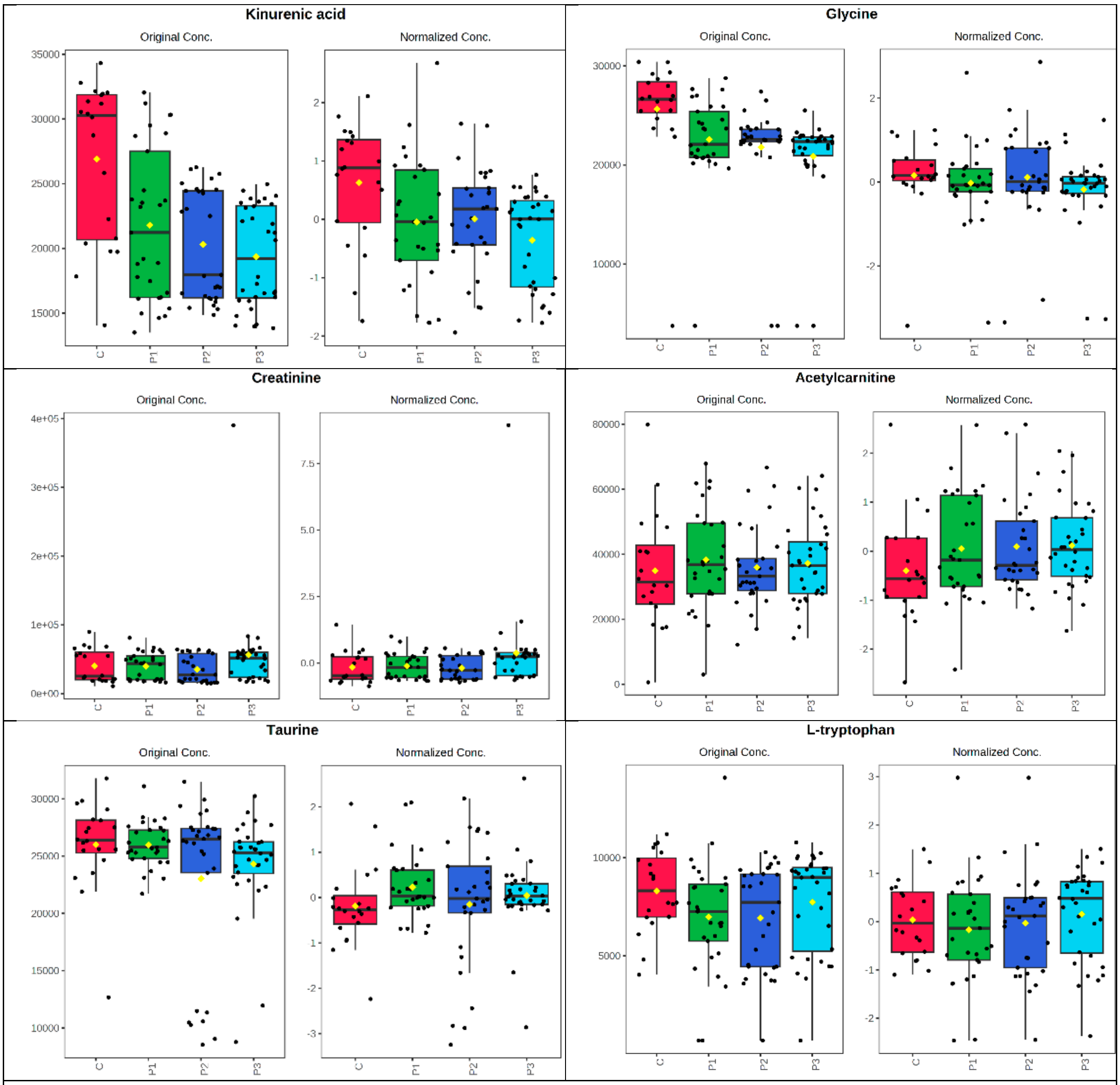

Figure S1. Graphic representation of the differences (expressed in MS peak intensities) between the groups C, P1, P2 and P3, for each of the serum potential biomarkers. The original and normalized (sample normalization by median values) are presented.

This representation reflects the decreasing levels of Glycine and Kynurenic acid vs gradual, slight increases of the other metabolites in the P1-P3 subgroups. The serum creatinine did not show significant modifications.

Fig. S2 shows graphically the differences (expressed in MS peak intensities) between the groups C, P1, P2 and P3, for each potential targeted biomarker in urine considering their original concentrations and the normalized ones by median values.

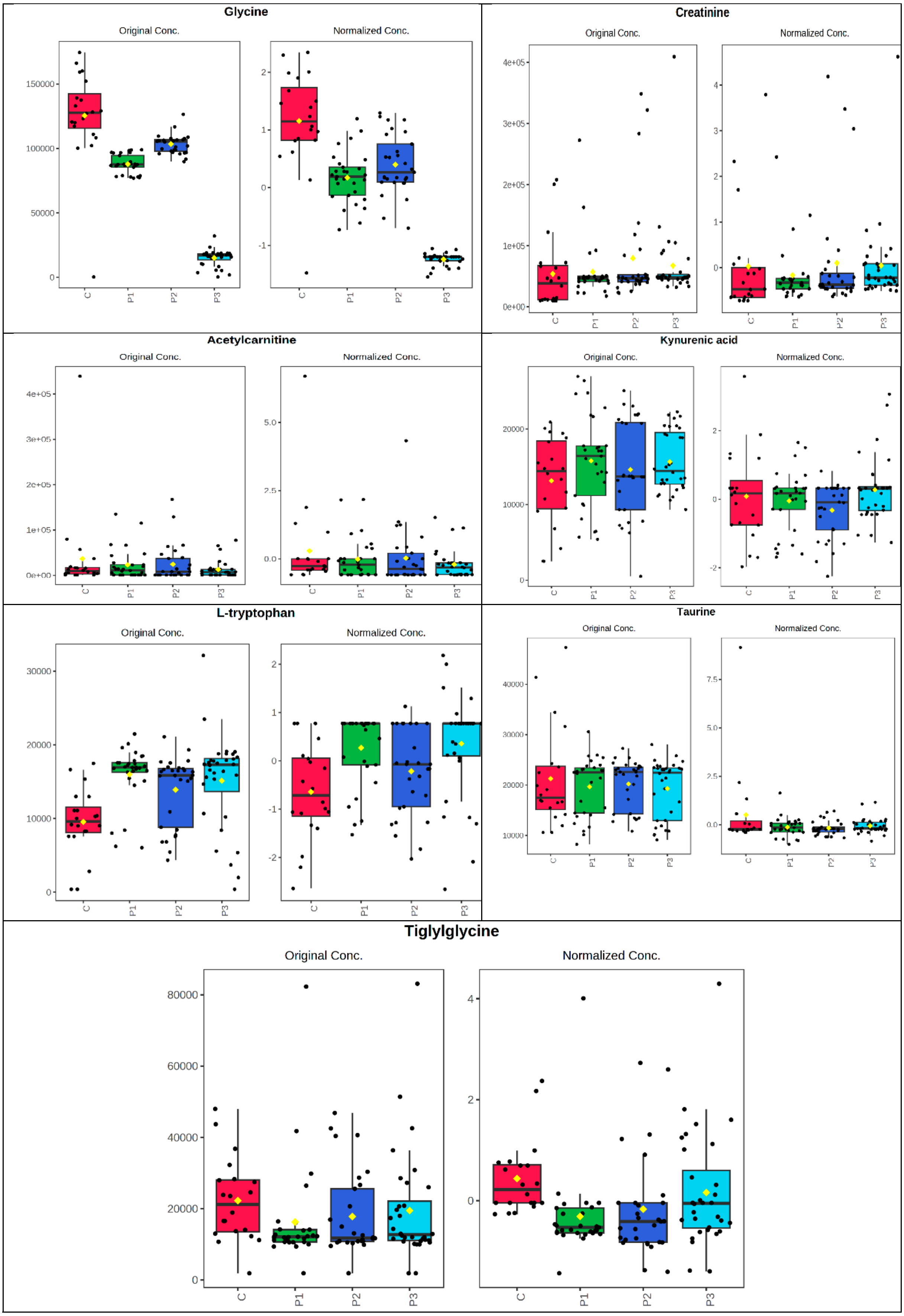
Glycine
Original Conc.
Normalized Conc.
150000
100000
50000
0
2
1
0
-1
C
P1
P2
P3
Creatinine
Original Conc.
Normalized Conc.
4e+05
3e+05
2e+05
1e+05
0e+00
4
3
2
1
0
Acetylcarnitine
Original Conc.
Normalized Conc.
4e+05
3e+05
2e+05
1e+05
0e+00
5.0
2.5
0.0
Kynurenic acid
Original Conc.
Normalized Conc.
20000
10000
0
2
0
-2
L-tryptophan
Original Conc.
Normalized Conc.
30000
20000
10000
0
2
1
0
-1
-2
Taurine
Original Conc.
Normalized Conc.
40000
30000
20000
10000
7.5
5.0
2.5
0.0
Tiglylglycine
Original Conc.
Normalized Conc.
80000
60000
40000
20000
0
4
2
0

Figure S2. Graphic representation of the differences (expressed in MS peak intensities) between the groups C, P1, P2 and P3, for each of the urine potential biomarkers. The original and normalized (sample normalization by median values) are presented.

This representation reflects the decreasing levels of Glycine, Taurine, increases of Tryptophan and Creatinine.

Fig.S3A shows the VIP Scores (less than 2.5) plot for the serum samples from groups C vs subgroups P1, P2, P3 of DKD patients and **Fig.S3B** the MDA scores (up to 0.4) showing the ranking of best predicted biomarkers by Random Forest analysis.

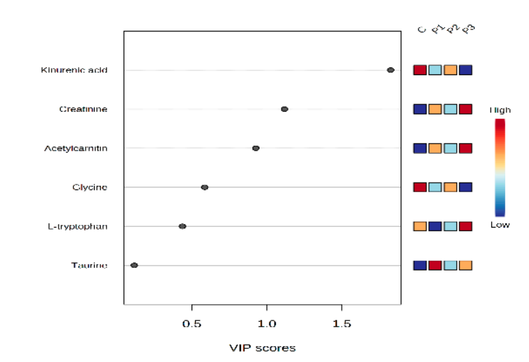

Figure S3A. VIP Scores plot for the serum samples from groups C *vs* subgroups P1, P2, P3 of DKD patients.

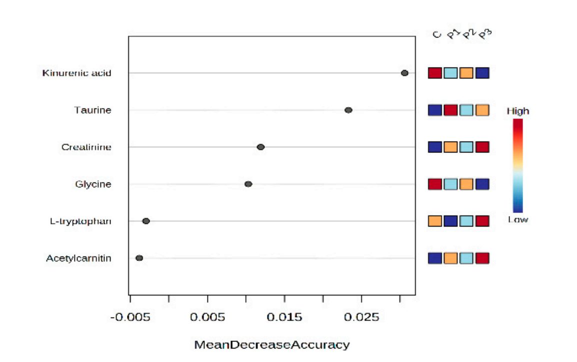

Figure S3B. Random Forest analysis: Mean decrease accuracy (MDA) scores showing the ranking of best predicted biomarkers in serum samples.

The VIP scores and MDA values confirm the gradual decreases of glycine and kynurenic acid from the healthy group to the subgroups P1, P2 and P3, while L-Acetylcarnitine and L-tryptophan showed opposite dynamics (gradual increases).

Fig.S4A shows the VIP Scores (less than 2.5) plot for the urine samples from groups C vs subgroups P1, P2, P3 of DKD patients and Fig.**S4B** the MDA scores (up to 0.4) showing the ranking of best predicted biomarkers by Random Forest analysis.

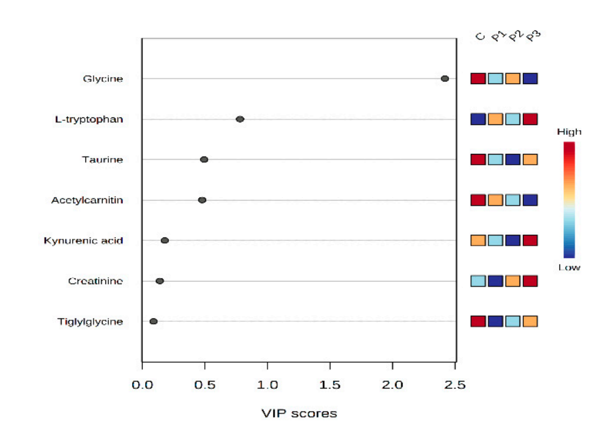

Figure S4A. VIP Scores plot for the urine samples from groups C *vs* subgroups P1, P2, P3 of DKD patients.

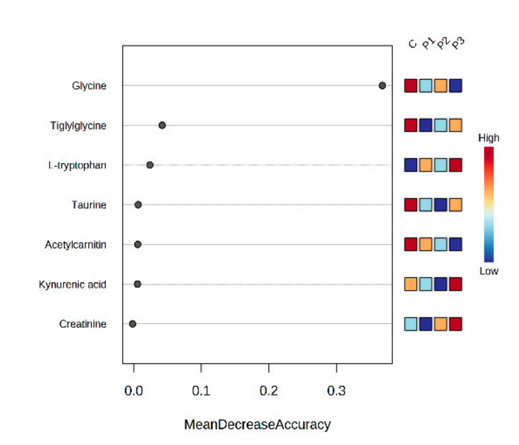

Figure S4B. Random Forest analysis: Mean decrease accuracy (MDA) scores showing the ranking of best predicted biomarkers in urine samples.

In urine, the VIP scores and MDA values confirm the decreases of Glycine, L-Acetylcarnitine, Taurine and Tiglylglycine from the healthy group to the subgroups P1, P2 and P3, while Creatinine, Kynurenic acid and L-tryptophan showed opposite dynamics (gradual increases).

Figure S5. Calibration curves for the selected putative biomarkers: creatinine (from blood serum and urine), Acetylcarnitine, Kinurenic acid, Glycine, Taurine, Tryptophan.

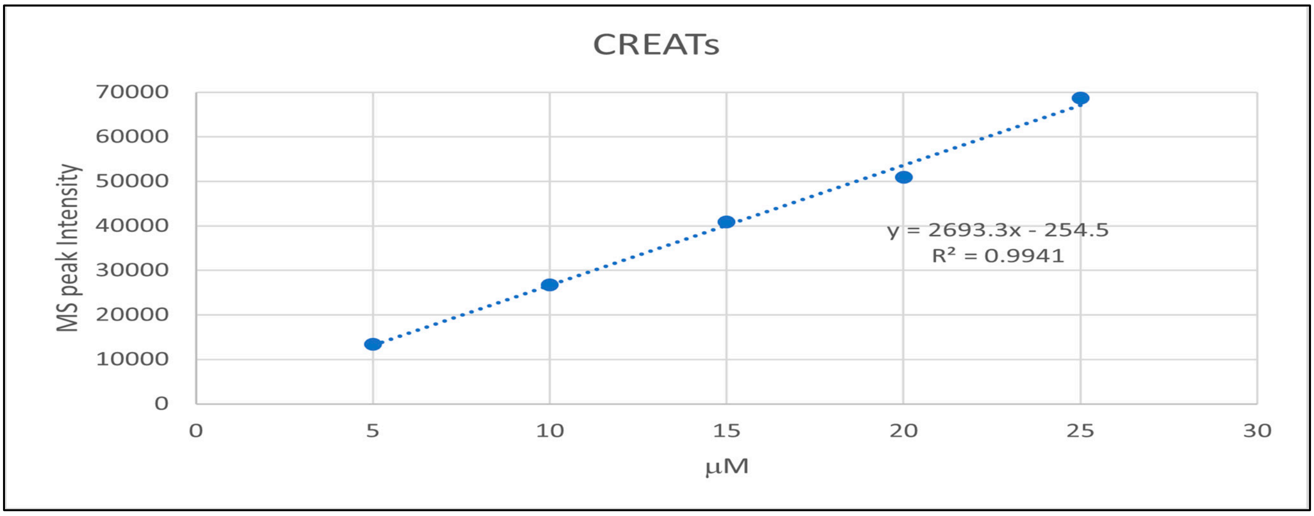

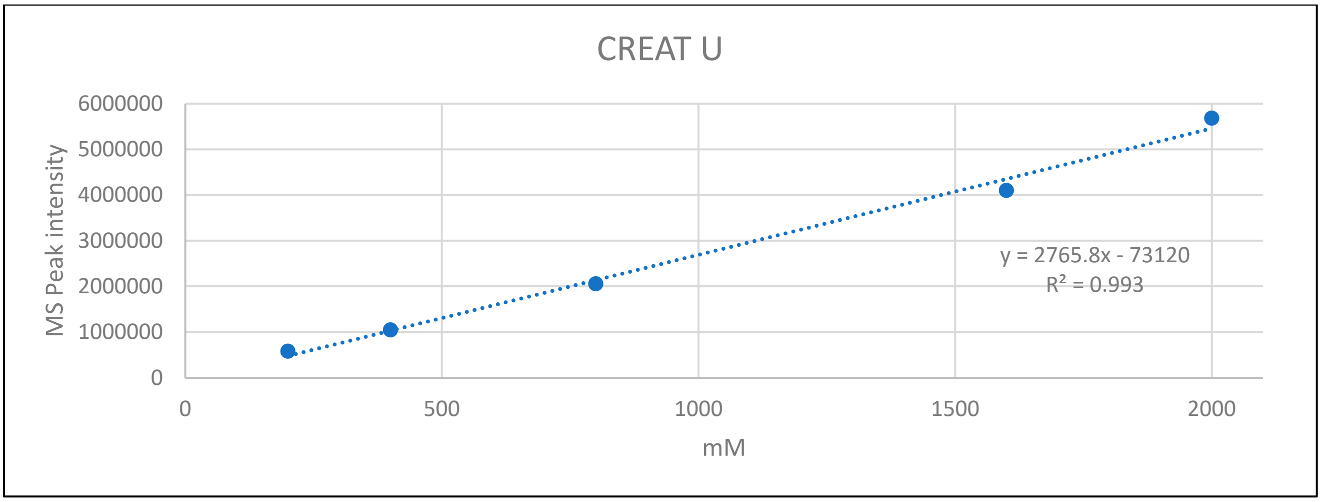

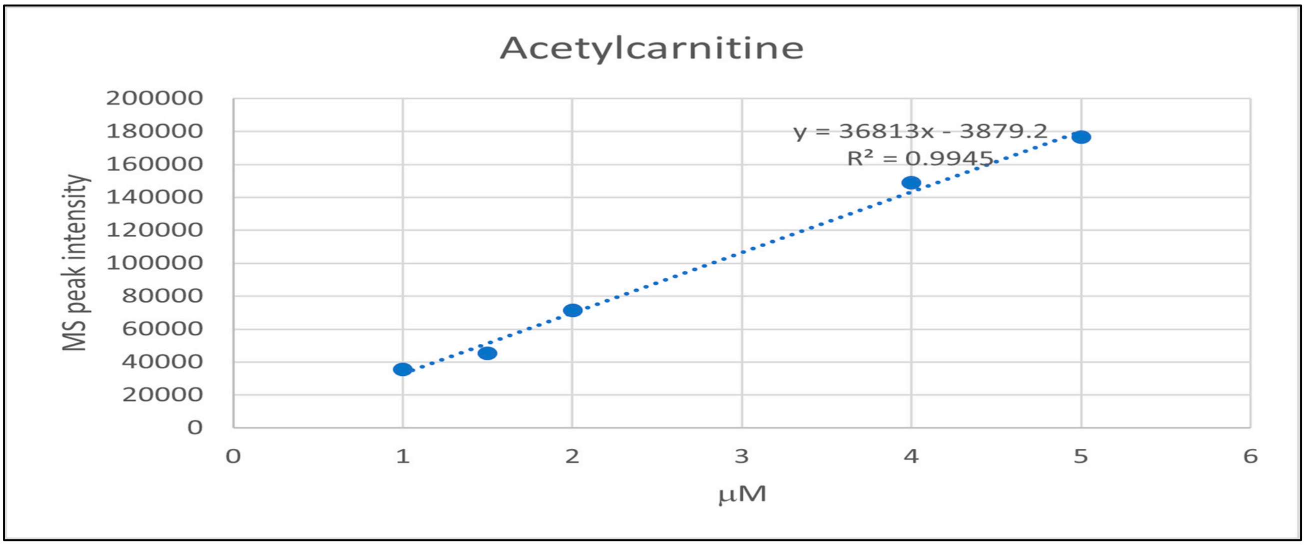
Acetylcarnitine
MS peak intensity
200000
180000
160000
140000
120000
100000
80000
60000
40000
20000
0
0
1
2
3
4
5
6
µM
y = 36813x - 3879.2
R² = 0.9945

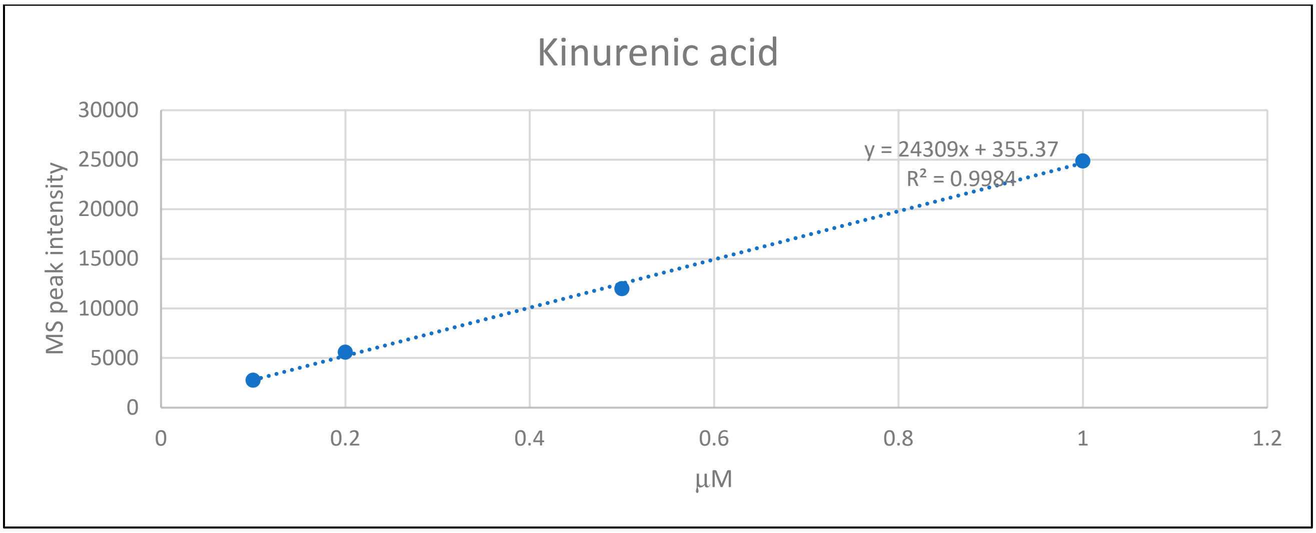
Kinurenic acid
MS peak intensity
30000
25000
20000
15000
10000
5000
0
0
0.2
0.4
0.6
0.8
1
1.2
µM
y = 24309x + 355.37
R² = 0.9984

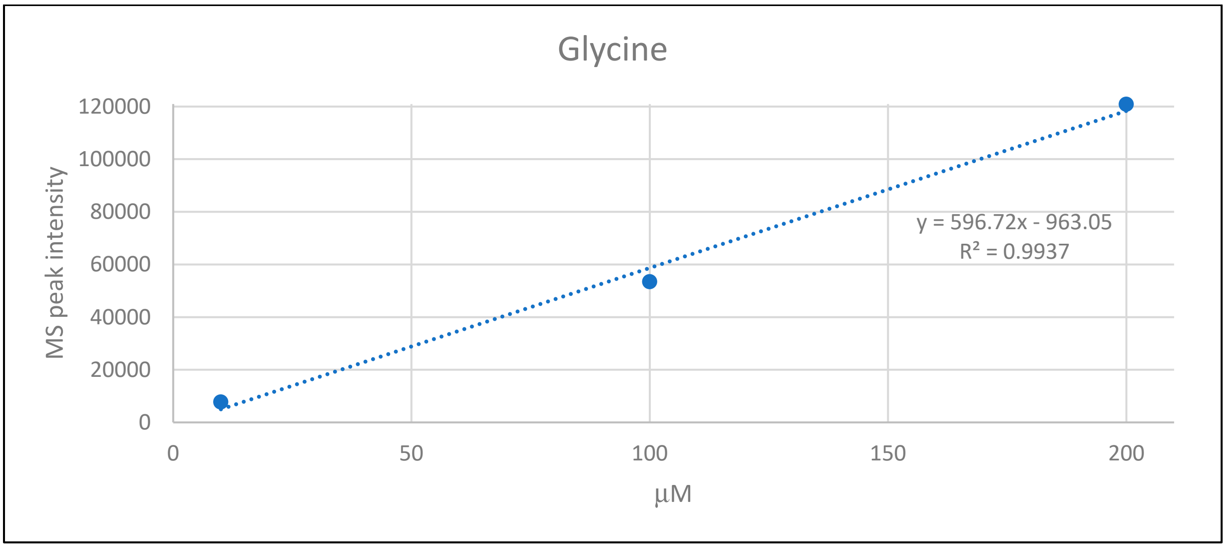
Glycine
MS peak intensity
120000
100000
80000
60000
40000
20000
0
0
50
100
150
200
µM
y = 596.72x - 963.05
R² = 0.9937

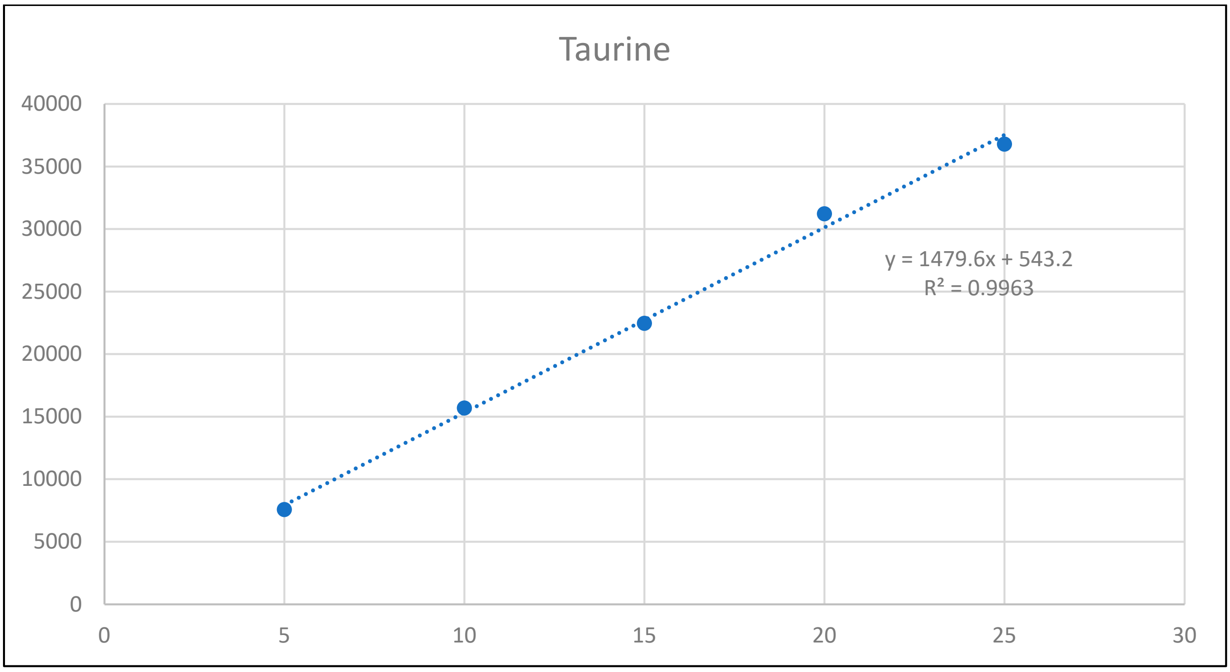

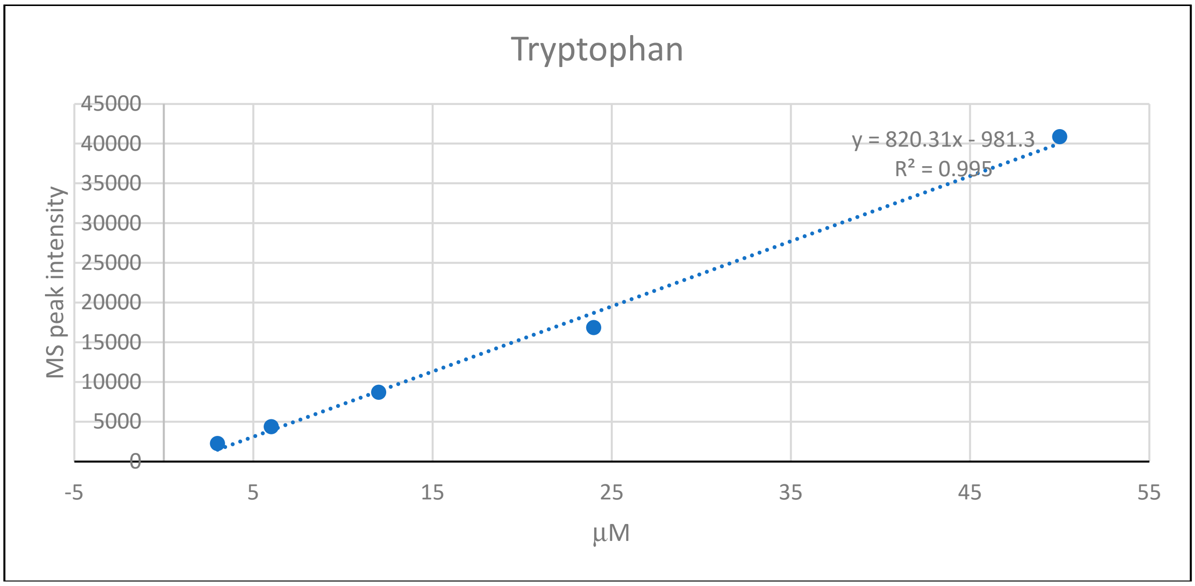

Table S1. Metabolites range

Biomarker	Normal range based on HMDB		Link
	Serum (µM)	Urine (µM/mM creatinine)	
L-tryptophan	40-90	3-6	https://hmdb.ca/metabolites/HMDB0000929
L-Acetyl carnitine	5-7	1-3	https://hmdb.ca/metabolites/HMDB0000201

Kynurenic acid	0.03 +/- 0.007<br><5	1-1.6	https://hmdb.ca/metabolites/HMDB0000715
Taurine	40-80	25-90	https://hmdb.ca/metabolites/HMDB0000251
Tiglylglycine	Not detected	0.1-7	https://hmdb.ca/metabolites/HMDB0000959
Creatinine	60.0-200	Large variation 6970 (3490-10500)-500-35000	https://hmdb.ca/metabolites/HMDB0000562
Glycine	120-450	106-135	https://hmdb.ca/metabolites/HMDB0000123

Table S2. Statiscal analysis

	C group	P1	P2	P3	Total	Test
N	20 (18.2%)	30 (27.3%)	30 (27.3%)	30 (27.3%)	110 (100.0%)	
**serum**						
Glycine	217 (200-229)	199 (169-204)	185 (169-199)	184 (170-187)	193 (171-204)	<0.001
Taurine	87 (83-93)	85 (82-90)	87 (76-91)	83 (78-87)	85 (80-91)	0.234
Kynurenic acid	6 (4-6)	4 (3-6)	4 (3-5)	4 (3-5)	4 (3-5)	<0.001
L-Acetylcarnitine	5 (4-7)	6 (4-7)	5 (4-6)	5 (4-6)	5 (4-6)	0.863
L-Tryptophan	57 (48-67)	51 (41-59)	52 (33-62)	48 (37-62)	51 (37-62)	0.164
**urine**						
Glycine	12 (9-14)	10 (7-14)	8 (6-12)	7 (5-13)	9 (6-13)	0.013
Taurine	8 (6-10)	9 (7-10)	8 (6-11)	9 (8-14)	9 (7-12)	0.204
Kynurenic acid	0 (0-0)	0 (0-1)	0 (0-0)	0 (0-1)	0 (0-1)	0.033
L-Acetylcarnitine	0 (0-0)	0 (0-0)	0 (0-1)	0 (0-1)	0 (0-0)	0.383
L-Tryptophan	7 (4-10)	4 (3-6)	12 (6-16)	16 (10-21)	9 (4-14)	<0.001
Tiglylglycine	5 (4-6)	4 (3-6)	4 (3-5)	2 (1-3)	4 (2-5)	<0.001
